# βc receptor antagonism mitigates sarcoidosis granuloma formation by targeting inflammatory signals and aberrant lipid metabolism

**DOI:** 10.3389/fimmu.2025.1733060

**Published:** 2025-12-16

**Authors:** Hao Wang, Damon J. Tumes, Simon Keam, Jia Wen Lim, Quynh Anh Nguyen, Ross Vlahos, Jonathan McQualter, Adam Quek, Carly Whyte, Harshita Pant, Timothy R. Hercus, Nicholas J. Wilson, Paul N. Reynolds, Catherine M. Owczarek, Angel F. Lopez, Steven Bozinovski, Kwok Ho Yip

**Affiliations:** 1 School of Health and Biomedical Sciences, RMIT University, Bundoora, VIC, Australia; 2 Centre for Respiratory Science & Health, Bundoora, VIC, Australia; 3 Centre for Cancer Biology, SA Pathology and the University of South Australia, Adelaide, SA, Australia; 4 CSL, Bio21 Institute, Parkville, VIC, Australia; 5 Adelaide Medical School, Faculty of Health and Medical Sciences, University of Adelaide, Adelaide, SA, Australia; 6 Department of Thoracic Medicine, Royal Adelaide Hospital, Adelaide, SA, Australia

**Keywords:** βc cytokines, granulomas, inflammation, lipid metabolism, macrophages, sarcoidosis

## Abstract

**Introduction:**

Sarcoidosis is a multisystem chronic inflammatory disorder of unknown etiology that primarily affects the lungs and currently has no cure. Macrophages are central to granuloma formation, and βc cytokines tightly regulate their activation and function. This study investigates the role of the βc receptor in granuloma development and evaluates βc antagonism as a potential therapeutic strategy for sarcoidosis.

**Methods:**

We utilized an *in vitro* model of human granuloma formation using sarcoidosis patient human peripheral blood mononuclear cells (PBMCs) and an *in vivo* vimentin-induced pulmonary sarcoidosis model in unique humanized βc transgenic (hβcTg) mice to assess the efficacy of βc antagonism in reducing granuloma formation and evaluate the underlying mechanism of action.

**Results:**

Anti-βc receptor antibody, CSL311, significantly reduced the formation of human granulomas from PBMCs exposed to purified protein derivative and decreased the pro-inflammatory cytokine production by the granulomas. Mechanistically, CSL311 inhibited hyperactivation of mTOR signaling and reduced lipid droplet formation in granuloma macrophages. In hβcTg mice challenged with vimentin, CSL311 effectively reduced both granuloma size and immune cell infiltration in the lung. RNA sequencing analysis of lung tissue further showed that CSL311 treatment suppressed the activation of vimentin-induced inflammatory, fibrotic, and lipid metabolic pathways.

**Conclusion:**

We identified that βc cytokines are critical regulators in driving inflammatory and metabolic processes that lead to granuloma formation in sarcoidosis. Precisely targeting the βc receptor effectively disrupts these pathogenic networks and offers a promising new strategy for mitigating sarcoidosis immunopathology.

## Introduction

Sarcoidosis is a chronic inflammatory disease of unknown cause characterized by the infiltration of myeloid and lymphoid cells, followed by granuloma formation, and ongoing tissue damage that results in fibrosis. Estimates suggest a prevalence of 1 to 16 cases per 10,000 people, with more than 90% of patients showing thoracic disease, including cases of isolated lymphadenopathy (1). Approximately 30% of individuals may show extrapulmonary manifestations, which can appear in various forms, including effects on the skin, eyes, liver, bone marrow, and central nervous system. Early diagnosis and treatment are essential to prevent progressive inflammation and fibrosis, which can lead to irreversible organ damage (2, 3). The management of sarcoidosis continues to evolve, although the best treatment approach has yet to be clearly identified. Traditionally, corticosteroids have been seen as the main treatment for individuals with severe symptoms, progressive pulmonary involvement, or significant extrapulmonary manifestations. However, many patients need more aggressive steroid-sparing immunosuppressive therapies or antifibrotic agents (4, 5). While methotrexate and infliximab can reduce the need for high-dose steroids, it is important to recognize their potential risks of broadly and irreversibly suppressing the immune system, which can lead to an increased risk of infection and liver damage (6, 7).

Pro-inflammatory myeloid cells, particularly macrophages, play a crucial role in driving the inflammatory response, granuloma formation, and subsequent fibrosis. Macrophages are central to the initial clustering that forms into granulomas in multiple organs. Upon exposure to, an as yet unidentified antigen, macrophages begin to cluster, and develop into epithelioid cells and multinucleated giant cells (MGCs). Surrounding these macrophage structures are lymphocytes, especially CD4^+^ T cells and neutrophils, which collectively contribute to the granulomatous pathology (8, 9). Macrophages express receptors for, and can be readily activated by, Granulocyte-Macrophage Colony-Stimulating Factor (GM-CSF) and Interleukin-3 (IL-3), which together with IL-5, comprise the βc family of cytokines. By sharing a common receptor subunit, βc, these cytokines demonstrate overlapping effects on macrophage activity (10). GM-CSF can act independently or in conjunction with other cytokines, such as interferon-gamma (IFN-γ) and tumor necrosis factor-alpha (TNF-α), to activate macrophages and coordinate the inflammatory response (11). Notably, IL-3, along with IFN-γ, can induce the fusion of macrophages into MGCs, a distinctive feature of granuloma formation (12). Additionally, elevated levels of IL-5, which are associated with pulmonary fibrosis, were detected in the serum of patients with fibrotic pulmonary sarcoidosis, although the cellular target is not yet clear (13). These observations present a valuable opportunity for therapeutic intervention in sarcoidosis.

Antagonizing the βc receptor could emerge as a powerful and effective strategy to modulate the hyperactive functions of various myeloid cells, which are predominantly driven by βc cytokines, while also preserving essential immune functions. An anti-βc receptor antibody, CSL311, which simultaneously blocks all three βc cytokine functions, has shown encouraging results in various humanized mouse models of immune diseases, where excessive activation of myeloid cells by GM-CSF, IL-3, and IL-5 contributes significantly to immunopathology (14–17). Furthermore, βc antagonism has demonstrated advantages over prednisolone in suppressing Th2 inflammation in a human airway tissue xenograft model (18).

In this study, we utilized a relevant *in vitro* preclinical model to investigate patient-derived human granuloma formation. More importantly, we developed an *in vivo* pulmonary sarcoidosis model using a unique human transgenic mouse expressing human βc receptor, which enabled the testing of potential new translational therapies. These comprehensive proof-of-principle studies demonstrated that the mechanism of βc receptor signaling contributes to granuloma formation and define βc receptor antagonism as a potential future immunotherapy option for sarcoidosis.

## Methods

### Study approval

This study received approval from the Central Adelaide Local Health Network Human Research Ethics Committee (Reference: 17814). The inclusion criteria for participants with sarcoidosis were established based on clinical features and fluorodeoxyglucose (FDG) PET/CT scans. All patients participating in this study are diagnosed with chronic sarcoidosis, exhibiting persistent symptoms for more than two years. These individuals display clinical manifestations associated with pulmonary conditions, and some additionally demonstrate manifestations in the cardiovascular and ocular systems. FDG PET/CT scans were employed for identifying patients with active disease (**Table 1**). Prior to their participation, all individuals provided informed written consent. For all *in vivo* experiments, female hβcTg mice aged 10 to 16 weeks were used, and all mice were bred in-house at the RMIT Animal Facility (Bundoora, Australia). Experiments were conducted in compliance with the ethical guidelines of the National Health and Medical Research Council of Australia and ARRIVE, with approval from RMIT University (ethics approval #AEC26548).

**Table 1:** Demographic characteristics of patients.

Age, yr [mean ± S.E.M (Range)]	55.3 + 2.8 (28 – 74)
Sex, N(%)	
Female	5 (29%)
Male	12 (71%)
Disease Severity, N(%)	
Stable	9 (53%)
Active	8 (47%)
Organ Involvement, N(%)	
Lung	17 (100%)
Heart	2 (12%)
Others (Bones, eyes, skin)	3 (18%)
Widespread	2 (12%)

### *In vitro* human granuloma model

The *in vitro* model of granuloma formation associated with sarcoidosis has been thoroughly described in prior research and closely mirrors the molecular characteristics of granulomas observed in the tissues of individuals affected by sarcoidosis (19–21). Peripheral blood mononuclear cells (PBMCs) were isolated from blood samples of patients with sarcoidosis through Ficoll gradient separation. The isolated PBMCs, at a concentration of 2 x 10^6^ cells/mL, were seeded on collagen I plus fibronectin-coated wells and equilibrated in RPMI media supplemented with 10% human AB serum (Sigma-Aldrich, US) and Penicillin-Streptomycin (Gibco) for 30 minutes at 37°C in an incubator with 5% CO_2_. Subsequently, the PBMCs were stimulated with either purified protein derivative (PPD, AJ Vaccines, Denmark)-coated beads (Polysciences, US) or uncoated beads. Granuloma size was evaluated through light microscopy on day 4. Following this evaluation, human monoclonal anti-βc antibody CSL311 (100 µg/mL) or isotype control antibodies (BM4, 100 µg/mL) were added to the appropriate treatment groups, and 50 µL of human AB serum was added to each well. This antibody treatment was repeated on day 5. On day 7, after assessing the granuloma size, the supernatants were collected, snap-frozen in liquid nitrogen, and subsequently stored at -80°C. The cells were then harvested for immunofluorescence staining or RNA extraction.

### Granuloma area and size assessment

Bright field microscopy images (100x magnification) were captured using a Nikon Eclipse Ts2 inverted microscope (Nikon, Japan). The granuloma area in each image was measured using Fuji ImageJ (1.54p, National Institutes of Health). Two experimenters were assigned the responsibility of independently capturing and analyzing images, ensuring a rigorous and objective approach to the analysis. Total granuloma area was presented as a percentage of the image capture area. For each condition in every independent experiment, we evaluated a minimum of ten fields of view. The data are presented as biological replicates.

### RNA isolation, cDNA preparation and real-time PCR

RNA from PBMCs (2 x 10^6^ cells) or granuloma cultures was extracted using TRIzol reagent (Life Technologies, USA) according to the manufacturer’s instructions. 1 µg of RNA was used for complementary DNA (cDNA) synthesis using the QuantiTect reverse transcription kit (QIAGEN, Netherlands). Quantitative real-time PCR was performed using a 1:5 dilution of cDNA with the QuantiTect SYBR Green PCR System (QIAGEN) and specific primers (**Supplementary Table S1**) on a Rotor-Gene 6000 PCR machine (QIAGEN). PCR assays were performed for 50 cycles (95°C for 15 s, 60°C for 30 s, and 72°C for 30 s). ct values were obtained from the Rotor-Gene Series 6000 Software (QIAGEN), and relative expression levels of mRNA were normalized to GAPDH or 18s RNA control and expressed using the 2^-ΔΔCt^ method.

### Immunofluorescence staining

Cells grown on coverslips were carefully washed with PBS. Sections were blocked in normal goat serum in CAS-Block (blocking buffer, Thermo Fisher Scientific, USA) for at least 30min in a humidified chamber at room temperature before mouse anti-CD68 antibodies (Invitrogen USA, KP1, 1:200 in blocking buffer) were added to the section and incubated overnight at 4°C. Sections were washed in distilled water, and Alexa Fluor^®^-555 goat anti-mouse secondary antibody (Thermo Fisher Scientific USA, 1:400 in blocking buffer) was added to the section and incubated for 1h at room temperature in the humidified chamber in the dark. Sections were washed in distilled water and incubated with BODIPY™ 493/503 (Thermo Fisher Scientific USA, 1 µM) in PBS for 20 min. After a final wash with distilled water, sections were mounted in Fluoroshield mounting medium with DAPI (Abcam, USA). Images were acquired using 40x (PlanApo 40x/1.0 W DIC) objective on a Zeiss LSM 700 confocal system and recorded using Z.E.N. 2011 (Black Edition) software (Carl Zeiss Microscopy, Germany). To ensure unbiased quantification, a second experimenter anonymized the images before the analysis was conducted using Fuji ImageJ (1.54p, National Institutes of Health), with thresholding applied to the corresponding no primary antibodies or isotype control. For each condition in every independent experiment, we evaluated a minimum of three fields of view. The data are presented as biological replicates.

### Cytokine release measurements

To assess the cytokines released by granulomas, we measured the supernatants collected using an ELISA kit for human GM-CSF, human IFN-γ, human TNF-α, and human IL-1β (eBioscience™) following the manufacturer’s instructions. Each sample was measured in duplicate, and the absorbance was recorded using an Epoch microplate reader (BioTek Instruments, USA).

### Immunoblot

Granulomas were collected and centrifuged at 180g for 5 min at 4°C. Cells were then lysed in 100 µl ice-cold lysis buffer containing 50 mM Tris-base, 100 mM NaCl, 5 mM EDTA, 10 mM Na4P2O7, 1% Triton X-100, 2 mM NaF, 1X complete protease inhibitors cocktail (Roche). Total cell lysates were separated with SDS-polyacrylamide gel electrophoresis (SDS-PAGE) and transferred to nitrocellulose membranes. Membranes were blocked in 5% non-fat dry milk in Tris-buffered saline that contained 0.1% Tween buffer; they were then probed with antibodies raised in rabbit against the phosphorylated form of mTOR(Ser2448, #5536), p70-S6K (Thr389, #9234) and 4E-BP1 (Thr37/46, #2855) at 1:1000 dilution. Membranes were then probed with horseradish peroxidase (HRP)–conjugated antibody against rabbit IgG (1:3000 dilution), and bands were visualized with ECL reagent (Bio-Rad Laboratories) with a LAS4000 imaging system (Fujifilm). Membranes were then stripped and re-probed with antibodies against the total form of mTOR (#2983) and β-actin (#4967) at 1:1000 dilution. All antibodies used were from Cell Signaling Technology. The band intensity was quantified using Fuji ImageJ (1.54p, National Institutes of Health), and data were normalized to the total amount of mTOR or β-actin, then to the untreated control.

### Purification of recombinant mouse vimentin

Expression and purification of recombinant mouse vimentin was carried out according to (22) with several modifications. A cDNA encoding recombinant mouse vimentin (NCBI RefSeq: NP_035831.2), comprising residues 2–466 fused to an N-terminal 8×His tag, was cloned into the pET41a expression vector (Merck-Millipore, Germany). The construct was transformed into *E. coli* ClearColi™ cells (Research Corporation Technologies, USA) for LPS-free protein expression. Following overnight expression at 16°C, vimentin predominantly accumulated in inclusion bodies. Inclusion bodies were washed with buffer containing 50 mM Tris-HCl (pH 8.0), 50 mM NaCl, 0.5% Triton X-100, and 1 mM DTT, then solubilized in 50 mM Tris-HCl (pH 8.0), 50 mM NaCl, 8 M urea, and 1 mM DTT. The solubilized protein was purified using IMAC followed by anion exchange chromatography. Refolding was achieved by stepwise reduction of the urea concentration. The refolded vimentin was stored frozen at -80°C in 1x DPBS (Lonza, Switzerland) containing 2 mM TCEP. For immobilization, vimentin was first bound in its denatured state to sterilized Ni-NTA agarose beads (30–40 µm; Cube Biotech, Germany) in PBS supplemented with 8 M urea and 2 mM TCEP. On-column refolding was then performed using the method described above. The immobilization efficiency was estimated at ~1 mg of vimentin per 100 µl of beads. The final product (His-Vim) was stored at 4°C as a 50% slurry in 1x DPBS (Lonza, Switzerland) containing 2 mM TCEP. Abbreviations: DPBS, Dulbecco’s Phosphate Buffered Saline; DTT, Dithiothreitol, IMAC, Immobilized metal affinity chromatography; LPS, Lipopolysaccharide; Ni-NTA, Nickel-Nitrilotriacetic acid; TCEP,Tris(2-carboxyethyl)phosphine.

### Vimentin-induced pulmonary sarcoidosis model

Female hβcTg mice were subcutaneously injected with 50 μg of free vimentin in conjunction with complete Freund’s adjuvant (CFA, Sigma-Aldrich, US) on day 0. Subsequently, the mice underwent an intravenous challenge with histidine-tagged vimentin bound to Ni-NTA agarose beads (His-Vim, ~40,000 beads), on days 9 and 12. To investigate the anti-βc signaling related to lung sarcoidosis, mice were randomly assigned to either the CSL311 or the isotype control treatment groups. The treatment involved intravenous administration (i.v., 50 mg/kg) to the vimentin-treated hβcTg mice on experimental days 0, 8, and 11. All mice were euthanized on experimental day 15 by intraperitoneal injection of pentobarbitone (1,625 mg/kg) to evaluate lung inflammation and pathology. Details on the allocation of animals to different analyses are provided in **Supplementary Table S2**.

### Histopathological analysis

Lung lobes were fixed, paraffin-embedded, and coronally sectioned at 4 μm thickness. Tissue sections were stained with Hematoxylin and Eosin (H&E). Whole-slide imaging was performed using an Olympus Slide Scanner (Olympus, Japan). Lung granulomas associated with the vimentin-bound beads were manually counted. The number of lung granulomas per mm² of tissue area was calculated as the number of granulomas identified on a given lung section divided by the lung section area. CellSens software (Olympus, Japan) was used for morphometric assessments of mean area (µm²) of detected granulomas. A region of interest (ROI) was manually defined to delineate the granuloma boundary, and the total area and Circle Equivalent Diameter of each granuloma were quantified using automated image analysis.

### BAL and tissue collection

To measure lung inflammatory cells, BAL was performed. Total cells recovered from the BAL were enumerated using a hemocytometer. Cytospins were prepared and stained with a Hemacolor^®^ Rapid staining kit (Merck Millipore, USA) for differential cell quantification. BAL cells were pelleted by centrifugation and snap-frozen in liquid nitrogen prior to -80 °C storage. Lungs were perfused free of blood with ice-cold PBS. Lung lobes were snap-frozen in liquid nitrogen prior to -80 °C storage and subsequent RNA-sequencing analysis.

### RNA-sequencing on mouse lungs

RNA was extracted from crushed frozen lung tissue using a RNeasy Plus kit (Qiagen, Germany) according to manufacturer’s instructions. The extracted RNA was subsequently used for bulk RNA-seq by the Australian Genome Research Facility (AGRF, Melbourne, Australia). RNA purity and integrity were assessed using an Agilent TapeStation System (Agilent Technologies, US) prior to library construction with the Illumina Stranded mRNA Prep kit (Illumina, US). Twenty million 150-bp paired end reads were performed on the Illumina NovaSeq X plus platform, and primary sequence data was then generated with the Illumina DRAGEN BCL Convert 07.021.645.4.0.3 pipeline. The raw sequencing data was analyzed in FastQC and trimmed using TrimGalore-0.6.7 to remove low-quality reads and adaptor sequences. The STAR aligner (v 2.7.4a) was used to map reads against the *Mus musculus* genome (Build version mm39) and featureCounts from the Subread package (v2.0.3) was used to generate the raw gene counts. Low gene counts were removed in ‘edgeR (v 3.38.4)’, followed by the trimmed mean of M-values (TMM) normalization and differential gene analysis. Differentially expressed genes (DEGs) were defined as having a false discovery rate (FDR) < 0.05. DEGs were visualized using ‘ggplot2 (v 3.4.4)’ and ‘VennDiagram (v 1.7.3)’. Gene Set Enrichment Analysis (GSEA) and Gene Set Variation Analysis (GSVA) were conducted using established gene sets from the Human Phenotype Ontology (HPO) database, the Kyoto Encyclopedia of Genes and Genomes (KEGG) database and the Molecular Signatures Database (MSigDB). ‘ClusterProfiler (v 4.10.1)’, ‘GSVA (v 1.50.5)’, ‘enrichplot (v 1.14.2)’ and ‘ggplot2’ were used for the ORA/GSEA/GSVA analysis and data visualization. RNA-seq data utilized in this study were from 6 control (SAL) mice, 5 vimentin-treated mice injected with isotype (VIM-ISO), and 5 vimentin-treated mice injected with CSL311 (VIM-CSL311).

### *In silico* human dataset analysis

Lung microarray datasets GSE16538 and GSE19976, as well as the skin RNA-Seq dataset GSE169146, were downloaded from the Gene Expression Omnibus (GEO). Datasets GSE16538 and GSE19976 were merged and inter-study batch effects were removed with ComBat using sva (v3.50.0) (23). Samples were categorized as normal (n=6), sarcoidosis (n=6), self-limiting sarcoidosis (n=8) or progressive sarcoidosis (n=7) as designated in the original papers. Differential expression was analyzed with limma (v3.58.1), and transcripts with FDR < 0.05 were deemed differentially expressed genes (DEGs). Gene set enrichment analysis (GSEA) was performed on pre-ranked DEG list using clusterProfiler (v4.10.1) and mapped against gene sets in MSigDB, KEGG and HPO database. Single-sample pathway activity was quantified by GSVA (v1.50.5), and core-enriched genes were visualized as row-scaled heatmaps using ComplexHeatmap (v2.18.0).

### Statistical analysis

All data were statistically analyzed using GraphPad Prism 10.0 (GraphPad, US) or R Studio. Data are presented as the mean ± SEM of biological replicates. Where detailed and appropriate, one-way ANOVA or two-way ANOVA with Tukey or Bonferroni post-tests was used for multiple comparisons. Asterisks above a group indicate that group’s level of significance relative to the control (*P <.05, **P <.01, ***P <.001). GSEA was conducted with ‘ClusterProfiler (v 4.22)’ (21). GSVA was performed using ‘GSVA (v1.50.5)’ (22).

## Result

### Increases in βc cytokine signaling signatures in sarcoidosis patients

**Figure 1:**
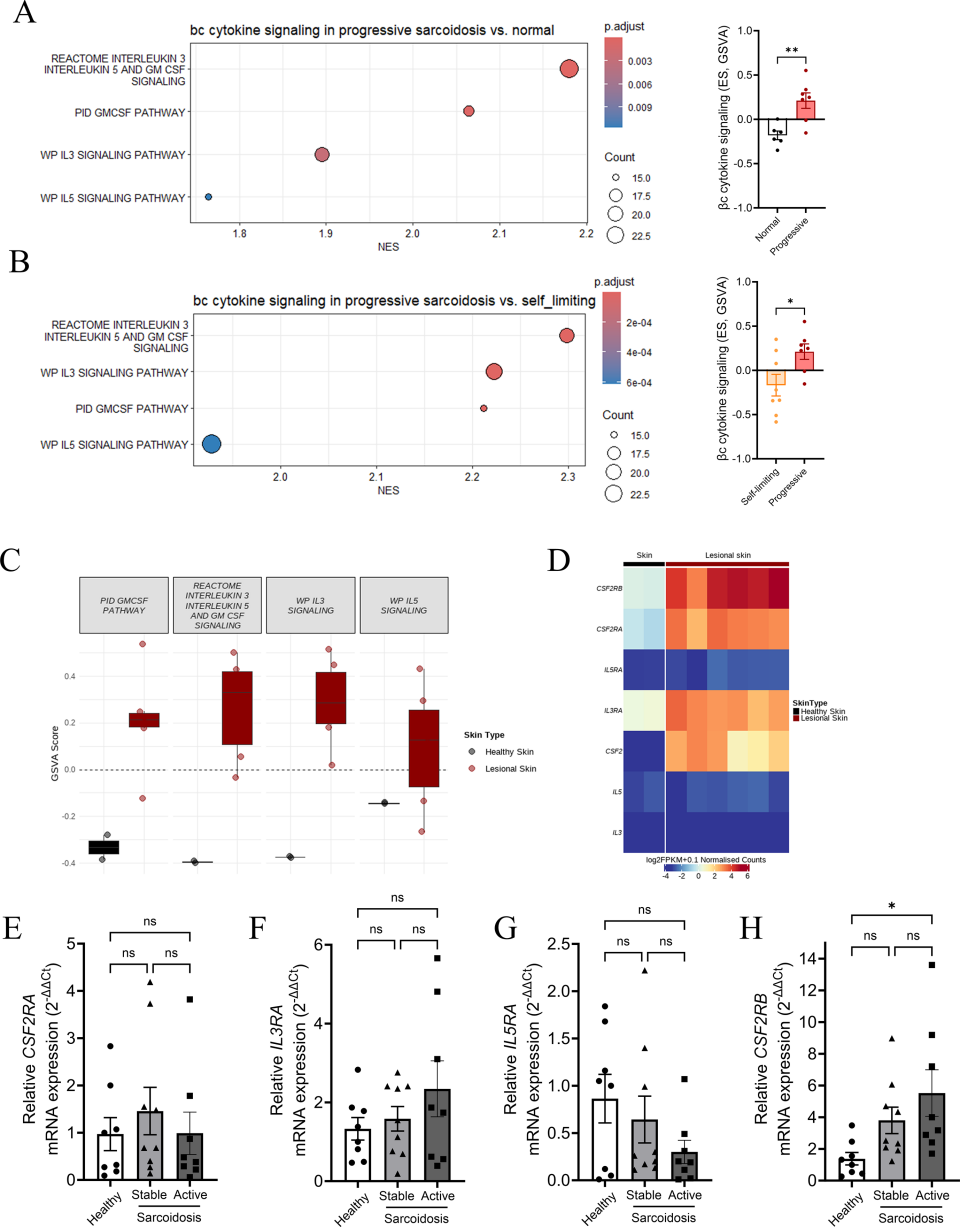
Enrichment of βc cytokine signatures in patients with sarcoidosis. **(A, B)** Gene set enrichment analysis (GSEA) was performed using gene sets from MSigDB to compare progressive sarcoidosis with both self-limiting sarcoidosis and normal lung tissue. Additional Single-sample GSVA (Gene set variation analysis) was conducted, with enrichment scores (ES) calculated for each sample. One-way ANOVA. **(C)** GSVA gene signature enrichment of βc activation pathways and associated functional signatures, and **(D)** Normalized expression levels of βc cytokine and receptor genes in normal and lesional skin of cutaneous sarcoidosis. **(E-H)** Transcriptional expression of βc cytokine receptors in PBMCs from healthy donor controls and sarcoidosis patients with stable or active diseases; Data are mean + S.E.M. from 7–9 donors in each group, One-way ANOVA with Bonferroni post-test.

To investigate βc cytokine signaling in sarcoidosis, we conducted an extensive analysis of 12 publicly available “omic” sarcoidosis datasets. This analysis encompassed both single-cell and bulk transcriptomics, to examine the relevance of the β-common pathway to the activity and severity of the disease. Notably, our findings identified a consistent correlation between the activation of the βc signaling pathway and granuloma samples from sarcoidosis patients across various organs (**Table 2**). We next performed an in-depth analysis of the transcriptomic datasets, comparing sarcoidosis lung tissues with normal lung tissues (GSE16538, PMID: 19218196) (24) and lung tissues from patients with nodular, self-limiting disease versus those with progressive, fibrotic disease (GSE19976, PMID: 20194811) (25). The two datasets were combined for co-analysis, and batch effects were removed. Gene set enrichment analysis (GSEA) revealed significant enrichment of βc cytokine signaling, both collectively and individually, in progressive sarcoidosis compared to self-limiting disease or normal lung tissue (**Figures 1A, B**). A heatmap analysis further demonstrated an upregulation of key genes involved in these pathways, particularly in the progressive sarcoidosis group, including *CSF2RB*, *CSF2RA*, *IL3RA*, *STAT5A*, and *STAT1* (**Supplementary Figure S1**). Single-sample gene set variation analysis (GSVA) further confirmed that the enrichment score (ES) for βc cytokine signaling was significantly higher in the progressive sarcoidosis group compared to the self-resolving group and normal controls (**Figures 1A, B**). Additionally, the cutaneous sarcoidosis dataset (GSE169146, PMID: 35668129) provided valuable insights by comparing skin samples from patients with those from a control group (26). The analysis highlighted a significant enhancement in the activation of all βc cytokine pathway signatures (**Figure 1C**). Furthermore, it revealed an increase in expression levels of specific receptor components, including *CSF2RB*, *CSF2RA*, and *IL3RA* in the diseased tissues (**Figure 1D**). These findings suggest that signaling activation via the βc receptor is associated with sarcoidosis and disease severity.

**Table 2:** *In silico* analysis on publicly available datasets on Sarcoidosis patient samples.

Sarcoidosis type	Samples	Platform	Dataset ID	Cohort comparison	Evidence for an increase in βc cytokine signalling
Pulmonary	BALC	mRNA array	GSE110777	24 Sarc	–
Pulmonary	BALC	mRNA array	GSE75023	15 Sarc/12 Ctl	++
Pulmonary	Lung tissues	mRNA array	GSE16538	6 Sarc/6 Ctl	++
Pulmonary	Lung Granuloma	RNA-Seq	GSE157671	6 Sarc/3 Ctl	+
Pulmonary	Lymph Granuloma	RNA-Seq	GSE157671	12 Sarc/3 Ctl	+
Pulmonary	BALC	RNA-Seq	GSE109516	22P/35NP	++
Pulmonary	Lung tissues	mRNA array	GSE19976	7P/8NP	+++
Cutaneous	Skin tissues	RNA-Seq	GSE169146	6 Sarc/2 Ctl	++++
Cutaneous	Skin tissues	scRNA-seq	GSE169147	3 Sarc/3 Ctl	+

BALC: bronchoalveolar lavage cells, Sarc: sarcoidosis patient, Ctl: health control, P: progressive sarcoidosis, NP: non-progressive sarcoidosis. ++++ All analyses evidence; +++ 75% of analyses demonstrating evidence; ++ 50% of analyses demonstrating evidence; + 25% of analyses demonstrating evidence;-No evidence.

We further validated the above findings in PBMC isolated from patients diagnosed with sarcoidosis. Our analysis revealed that the expression levels of the receptor α subunits were not altered across the groups. However, we observed a significant increase in βc receptor gene expression in PBMCs from sarcoidosis patients. Notably, PBMCs from those with active sarcoidosis exhibited the highest levels of βc receptor expression, suggesting a possible peripheral signature (**Figures 1E–H**). Furthermore, our comparative analysis revealed no significant differences in the mRNA expression levels of GM-CSF, IL-3, and IL-5 between healthy donors and patients with sarcoidosis (**Supplementary Figure S2**).

### βc receptor antagonism inhibited *in vitro* human granuloma formation

**Figure 2:**
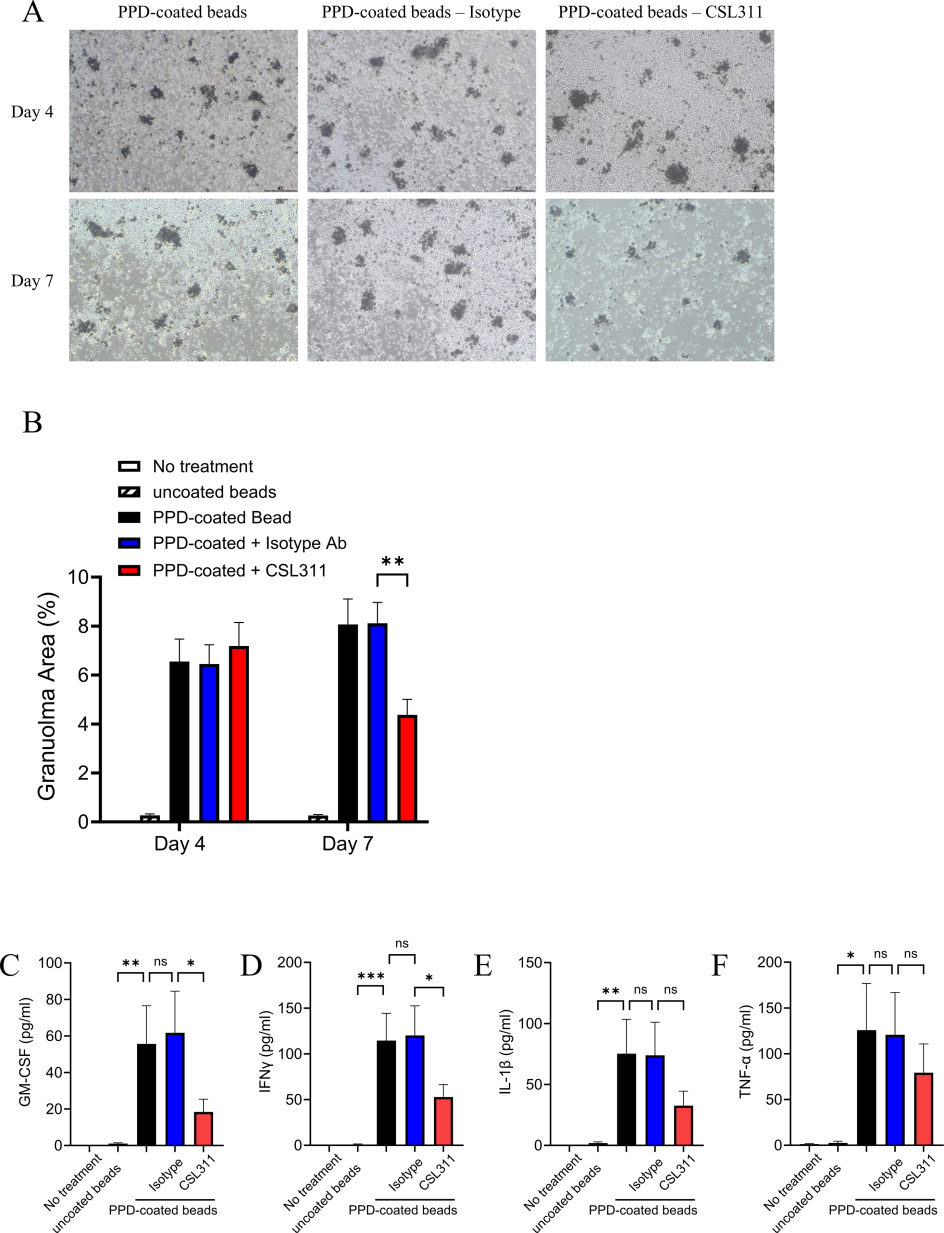
CSL311 inhibited *in vitro* granuloma-like aggregate formation induced by PPD-stimulated PBMCs. **(A)** Representative bright field microscopy images (100x magnification) were captured on days 4 and 7 after the incubation of PBMCs with PPD-coated or uncoated beads. The scale bar is 200 μm. **(B)** Quantitative measurement of granuloma area at days 4 and 7 under all treatment conditions. Data are mean + S.E.M. of 7 independent experiments. Two-way ANOVA with Bonferroni post-test. **(C-F)** Supernatants were collected after 7-day treatment. Levels of **(C)** GM-CSF, **(D)** IFN-γ, **(E)** IL-1β and **(F)** TNF-α in the supernatant were measured by ELISA. Data are mean + S.E.M. of 7 independent experiments. One-way ANOVA with Bonferroni post-test.

In previously reported *in vitro* granuloma models, the incubation of PBMCs obtained from patients with sarcoidosis with tuberculin PPD-coated beads led to the formation of granuloma-like aggregates (19–21). This model is a well-established system for studying human granuloma formation *in vitro* and was utilized in this study to evaluate the effects of βc receptor antagonism on granuloma formation. Following a 4-day incubation with PPD-coated beads, we carefully examined the granuloma-like cell aggregates under an inverted microscope, which enabled us to confirm the presence of granuloma aggregates and effectively assess both their area and size. Following the formation of granuloma-like aggregates, we administered either CSL311 or isotype control antibodies. By day 7, we observed that treatment with CSL311 resulted in a significant reduction in the area of the aggregates compared to both the untreated and isotype-treated control groups (**Figures 2A, B**). Furthermore, the granulomas treated with CSL311 showed a marked decrease in size, resulting in a predominance of smaller cell aggregates at the time of assessment (**Supplementary Figure S3**). Additionally, as part of our prophylactic treatment strategy, we incubated PBMCs with CSL311 or an isotype control antibody prior to their exposure to PPD. This approach demonstrated that CSL311 nearly completely inhibited the initiation of granuloma formation (**Supplementary Figure S4**).

Granulomatous inflammation involves the regulatory effects of several cytokines produced by local mononuclear phagocytes, T cells, and other cells within the inflammatory microenvironment. In the context of sarcoidosis, granulomatous inflammation is characterized by the dominant expression of the critical cytokines, GM-CSF, IL-1β and T helper 1 (Th1) cytokines, including IFN-γ and TNF-α. In this study, we evaluated the levels of these cytokines in the culture supernatant on Day 7. Our data show that the *in-vitro* PPD-induced granulomas produced all these cytokines, which may further facilitate granuloma expansion. Importantly, we found that treatment with CSL311 significantly reduced the release of GM-CSF and IFN-γ, while also demonstrating a trend toward lower levels of IL-1β and TNF-α (**Figures 2C-F**).

### CSL311 suppressed mTOR activity and aberrant lipid droplet formation

**Figure 3:**
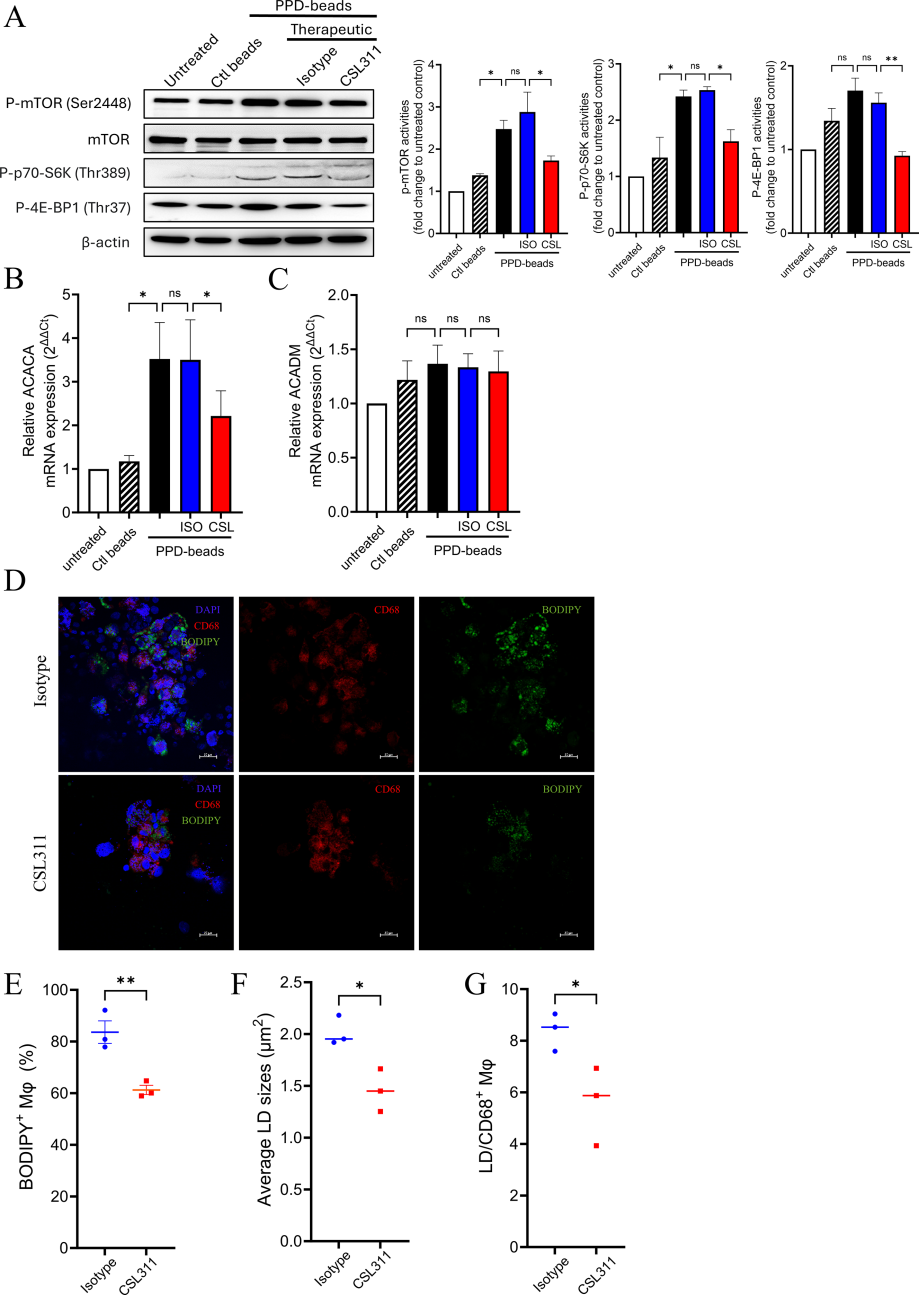
Treatment with CSL311 suppressed mTOR pathway activity and reduced aberrant lipid droplet synthesis in granulomas. **(A)***Left*, mTOR metabolic pathway analysis within granuloma cultures following a 7-day treatment with PPD-coated beads. Phosphorylation of (p-)mTOR, p70S6K, and p4E-BP1 signaling in whole cell lysates of the granuloma cultures was assessed. Data are representative of 3 independent experiments with similar results. Right, Quantification of immunoblot band intensities. Data are expressed as mean + S.E.M, One-way ANOVA with Bonferroni post-test. **(B, C)** Transcriptional level of **(B)** Acetyl-CoA Carboxylase (ACACA) and **(C)** Acyl-CoA Dehydrogenase (ACADM) in granuloma culture following a 7-day treatment with PPD. Data are mean + S.E.M. from 6 independent experiments. unpaired t-test. **(D)** Representative confocal immunofluorescence images of granulomas (400x magnification) following a 7-day treatment with PPD. The granulomas were stained for anti-CD68 (red), BODIPY 493/503 (green) and 4’,6-diamidino-2-phenylindole (DAPI) (blue). The scale bar measures 20 μm. **(E)** Percentage of BODIPY positive macrophage, **(F)** average lipid droplet (LD) size, and **(G)** average LD present in each CD68+ macrophage were analyzed. These data mean ± S.E.M. of 3 independent experiments, student’s t-tests.

It is well-established that chronic signaling through the metabolic checkpoint kinase mTOR promotes macrophage granuloma formation and is an indicator of sarcoidosis progression (27). In this study, we assessed the activities of the mTOR kinase and its principal substrates, eIF4E-binding protein (4E-BP1) and p70 S6 kinase (S6K), utilizing cell lysates obtained from granulomas at Day 7. Immunoblot analysis demonstrated an increase in the phosphorylation of mTOR, 4E-BP1, and S6K in granulomas induced by PPD stimulation in comparison to untreated and control beads controls. Notably, treatment with CSL311 led to a reduction in mTOR pathway activity (**Figure 3A**).

Human sarcoid granulomas are characterized by the presence of lipid-laden CD68^+^ macrophages that exhibit aberrant lipid metabolism. The βc cytokines, particularly IL-3 and GM-CSF, play a crucial role in lipid metabolism and lipid droplet (LD) dynamics by activating mTOR signaling. We investigated transcriptional alterations in genes associated with lipid metabolism. We found that the expression of acetyl-coenzyme A carboxylase alpha (ACACA), which serves as the rate-limiting enzyme in *de novo* fatty acid synthesis, increased following PPD stimulation (**Figure 3B**). This upregulation was effectively inhibited by treatment with CSL311. In contrast, the expression of acyl-coenzyme A dehydrogenase (ACADM), an enzyme essential for the catabolism of medium-chain fatty acids, remained unaffected by PPD stimulation (**Figure 3C**). Further assessment of lipid droplet synthesis in CD68^+^ macrophages within granulomas was conducted using BODIPY staining techniques (**Figure 3D**). The results showed that treatment with CSL311 significantly reduced the proportion of BODIPY^+^ macrophages in the granulomas (**Figure 3E**). Additionally, both the average size of lipid droplets (**Figure 3F**) and their quantity within CD68^+^ macrophages (**Figure 3G**) were diminished post-treatment with CSL311, suggesting a significant impact on lipid metabolism associated with βc receptor antagonism.

### CSL311 reduced granuloma burden in an *in vivo* model of lung sarcoidosis

**Figure 4:**
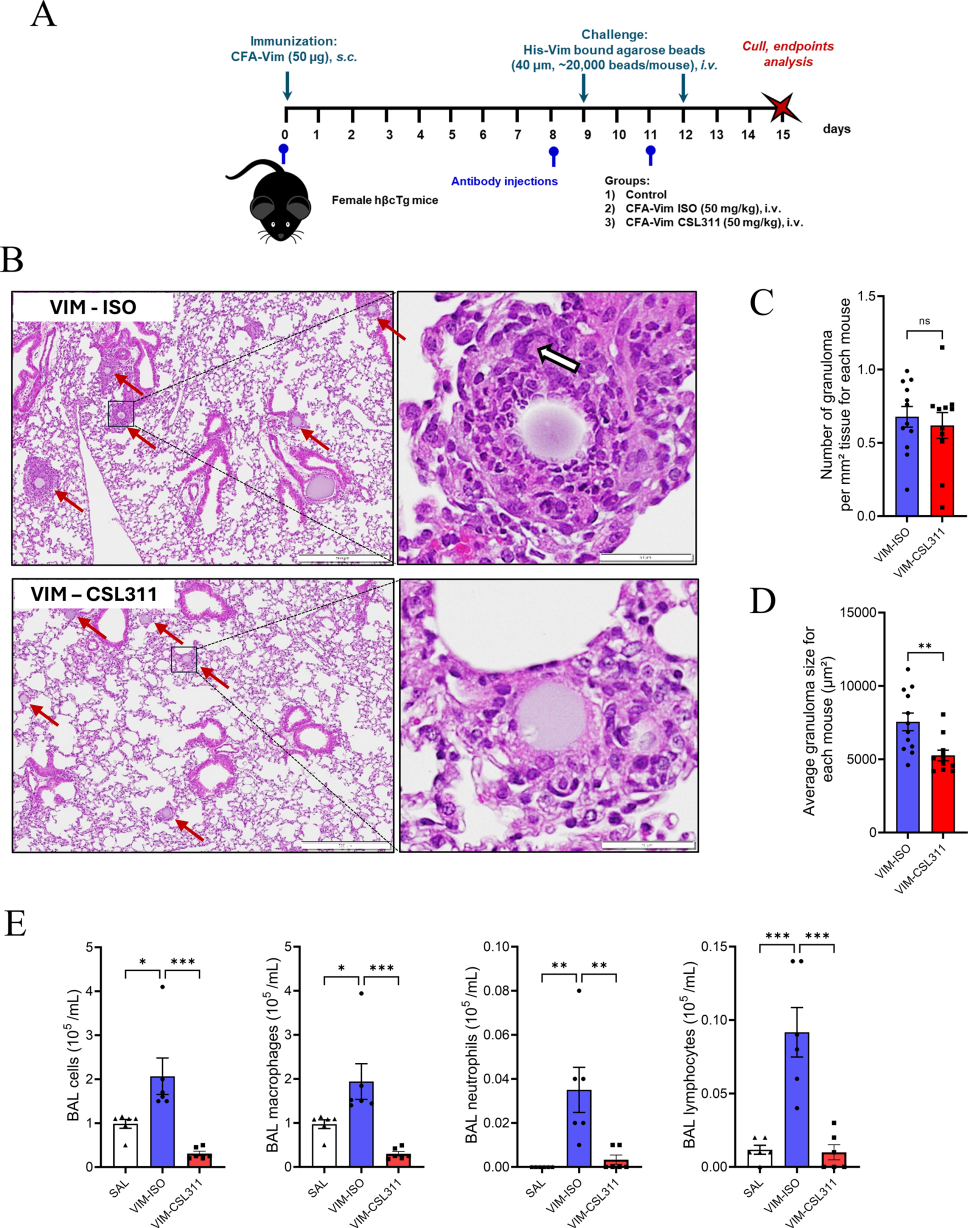
Blockade of βc cytokine signaling reduced lung granuloma in vimentin-immunized hβcTg mice. **(A)** Schematic of the vimentin-induced lung sarcoidosis model and the CSL311 treatment regimen (n=11-12). **(B)** Lung granuloma was analyzed on H&E-stained tissue sections. Scale bar is 500 µm in the original images and 50 µm in the inserts. Arrowhead shows an MGC. **(C)** The number of lung granulomas per mm² of tissue area **(D)** and the area (µm²) of granulomas in each mouse were analyzed. **(E)** The number of macrophages, neutrophils, eosinophils and lymphocytes in BAL fluid (n=6). Data are mean + S.E.M,; student’s t-tests or One-way ANOVA with Tukey post-test.

We subsequently conducted a pre-clinical model of lung sarcoidosis utilizing humanized hβcTg mice. These humanized transgenic (Tg) mice are characterized by the expression of the human (h) βc receptor (hβcTg) and the absence of murine βc/βIL-3 receptors, as previously described (16). The administration protocol, as previously illustrated, involved the subcutaneous injection of 50 μg of free vimentin, followed by an intravenous infusion of histidine-tagged vimentin bound to Ni-NTA agarose beads (His-Vim). To establish a proof-of-concept for βc receptor antagonism as a potential therapeutic strategy, we aimed to inhibit βc signaling by administering CSL311 to vimentin-treated mice (**Figure 4A**). It is important to highlight that this study did not include a group that received only Ni-NTA beads. Additionally, prior research showed that vimentin-beads alone elicited only a minimal level of granulomatous inflammation, and the addition of vimentin immunization markedly potentiated granuloma formation, supporting an antigen-specific effect from vimentin (22). Histopathological analysis conducted on day 15 revealed no granuloma formation in the saline control group (data not shown). In contrast, the vimentin-treated groups exhibited distinct lung granulomas (red arrowhead) along with the presence of multinucleated giant cells (MGCs) (**Figure 4B**. white arrowhead). Notably, while treatment with CSL311 (VIM-CSL311) did not result in a reduction in the total number of granulomas, it significantly decreased the average size of the granulomas compared to the isotype treatment group (VIM-ISO, **Figures 4C, D**). Furthermore, in the analysis of bronchoalveolar lavage (BAL) cellular components, CSL311 significantly reduced vimentin-induced total BAL cell counts, driven by reductions in BAL macrophages, neutrophils, and lymphocytes (**Figure 4E**).

**Figure 5:**
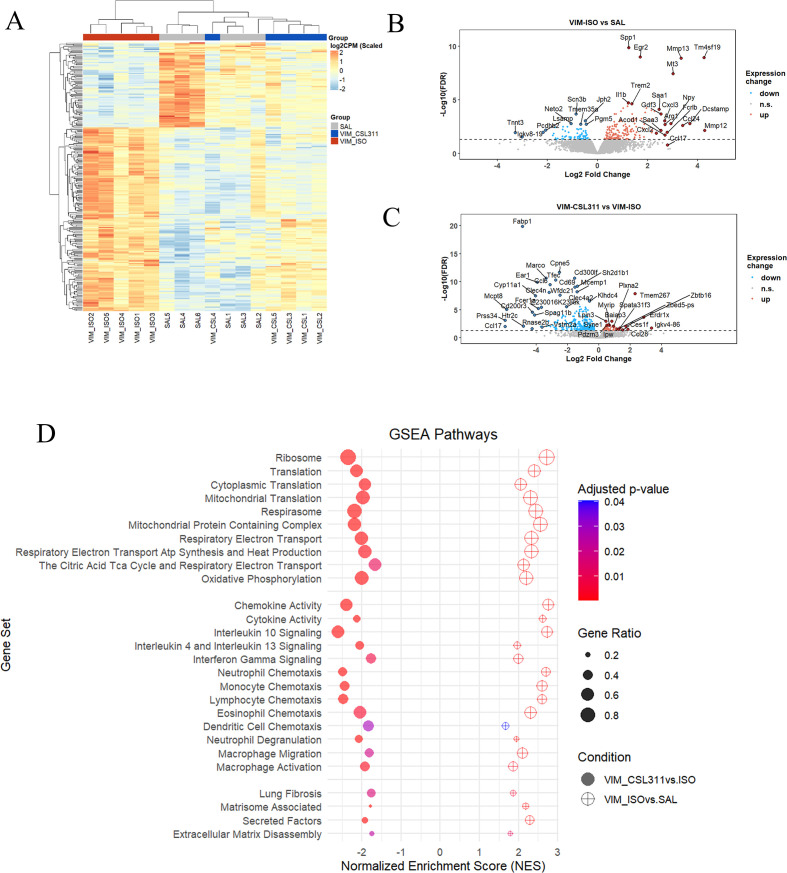
Treatment with CSL311 reversed vimentin-induced transcriptomic alterations in lung tissue. **(A)** Heatmap analysis to visualize the expression levels of all the vimentin-specific DEGs across all three groups. **(B, C)** Volcano plots to visualize the differential expression of genes between **(B)** the VIM-ISO and SAL groups and **(C)** the VIM-CSL311 and VIM-ISO groups. Upregulated genes are in red and downregulated genes are in blue. n=5-6. **(D)** A Broader pathway analysis was further performed using the MSigDB database.

To gain deeper insights into how βc receptor antagonism reduced granuloma burden at a molecular level, we conducted RNA-seq analysis of lung samples collected from the vimentin model. A heatmap analysis of vimentin-induced differentially expressed genes (DEGs) revealed distinct transcriptional profiles between saline controls (SAL group) and VIM-ISO group. Remarkably, VIM-CSL311-treated mice clustered closely with the SAL group, indicating that CSL311 treatment reversed vimentin-induced changes in gene expression (**Figure 5A**). Volcano plots highlighted key significantly upregulated and downregulated DEGs in VIM-ISO group compared to SAL group (**Figure 5B**) and VIM-CSL311 group compared to VIM-ISO group (**Figure 5C**). After vimentin-treatment (VIM-ISO vs SAL), genes associated with lung remodeling (*Spp1*, *Npy*, *Saa1* and *Saa3*), macrophage activation (*Mmp12*, *Mmp13*, *Arg1* and *Trem2*), inflammatory chemokines and cytokines (*Ccl24*, *Cxcl2*, *Cxcl3* and *Il1b*), and MGC formation (*Dcstamp*) were among the most significantly upregulated DEGs. Conversely, genes linked to tissue integrity and adhesion (*Pgm5*, *Pcdhb2*) were among the most downregulated DEGs (**Figure 5B**). Following CSL311 treatment (VIM-CSL311 vs VIM-ISO), genes associated with myeloid cell response (*Ccl6*, *Marco*, *Cd300lf*, *Tfec*, *Cd69*, *Clec4n* and *Clec4a2*), T cell response (*Ccl17* and *Sh2d1b1*), mast cell and eosinophil cell response (*Mcpt8*, *Mcemp1*, *Fcer1a*, *Prss34*, *Ear1*, and *Rnase2b*), and lipid metabolism (*Fabp1, Cpne5, Vism2a*) were significantly downregulated (**Figure 5C**). CSL311 treatment also affected the expression levels of several key genes associated with granuloma inflammation, immune cell infiltration into granulomas, fibrosis related to sarcoidosis, and lipid metabolism in foamy macrophages (**Supplementary Figure S5**). Next, a comprehensive pathway analysis was conducted utilizing the MSigDB database. In mice treated with vimentin, a significant upregulation of energy metabolic pathways was observed, including mitochondrial respiration, the tricarboxylic acid (TCA) cycle, and oxidative phosphorylation. Promisingly, these alterations were effectively reversed following treatment with CSL311. Additionally, the analysis highlighted the activation of inflammatory pathways associated with chemokine and cytokine signaling, particularly IL-4 and IL-13 signaling, in the disease state. The study also offered valuable insights into the chemotaxis of myeloid cells—specifically neutrophils, eosinophils, macrophages, and dendritic cells—as well as lymphocyte migration. Treatment with CSL311 demonstrated a significant capacity to suppress key inflammatory pathways (**Figure 5D**). Additional findings indicated that pathways related to tissue remodeling, particularly those implicated in lung fibrosis, matrisome-associated genes, and secreted factors, were significantly upregulated in the disease condition. These pathways were subsequently attenuated through treatment with CSL311 (**Figure 5D**).

**Figure 6:**
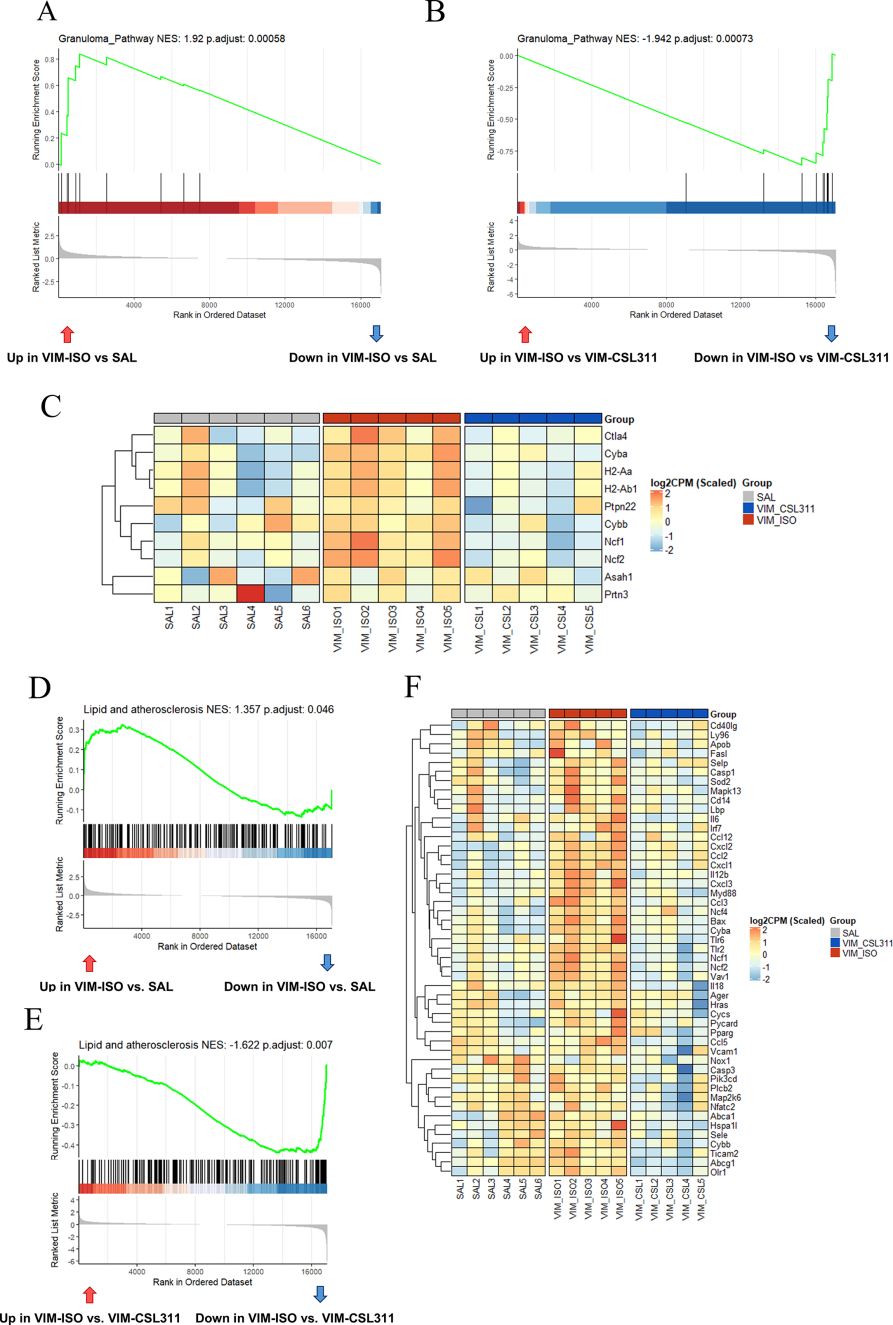
Reduction of the Granuloma-associated pathway, and the Lipid and Atherosclerosis pathway changes by CSL311. **(A, B)** GSEA using the Granulomatosis gene set for comparisons between VIM-ISO vs. SAL and VIM-CSL311 vs. VIM-ISO was performed. **(C)** The expression level of the member genes from Granulomatosis (HP:0002955) was visualized in a heatmap across all three groups. **(D, E)** GSEA using the KEGG Lipid and Atherosclerosis pathway on VIM-ISO vs. SAL and VIM-CSL311 vs. VIM-ISO comparisons was performed. **(F)** The expression level of the core enrichment genes from the lipid metabolism and atherosclerosis pathway was visualized in a heatmap across all three groups.

Since sarcoidosis is characterized as a granulomatous disease, we analyzed the pulmonary sarcoidosis patient dataset, utilizing the Granulomatosis HP gene set from the Human Phenotype Ontology (HPO) database. This gene set offers a comprehensive framework for understanding the various pathological processes involved in granuloma formation. Our analysis revealed that genes related to human granulomatosis were markedly upregulated in progressive sarcoidosis tissue compared to both normal tissue (NES=1.868) and tissue from patients with self-limiting disease (NES=1.927) (**Supplementary Figures S6A, B**). Following this, we applied the mouse homologs of the Granulomatosis gene set to analyze our mouse RNA-seq data using GSEA. As depicted in **Figure 6A**, we found that genes associated with this pathway were significantly upregulated in vimentin-induced sarcoidosis compared to saline controls (VIM-ISO vs SAL, NES=1.92). Moreover, our RNA-seq analysis in mice further demonstrated that treatment with CSL311 effectively reduced the expression of genes associated with the Granulomatosis gene set (VIM-CSL311 vs VIM-ISO, NES=-1.942) (**Figures 6B, C**). Our analysis also revealed valuable insights, indicating that pathways related to lipid metabolism and atherosclerosis were activated in the vimentin model (**Figure 6D**), aligning well with findings from human sarcoidosis patient datasets (**Supplementary Figures S6C, D**). Importantly, we observed that the lipid metabolism pathway was downregulated following treatment with CSL311 (**Figures 6E, F**). This change is consistent with the results from the *in vitro* model, where we observed a significant reduction in lipid droplet formation in granuloma macrophages following CSL311 treatment. These findings suggest potential therapeutic avenues that warrant further exploration.

## Discussion

Sarcoidosis is a multifaceted disease that presents significant treatment challenges, underscoring the need for effective and targeted management strategies. Central to the formation of granulomas are macrophages, which serve not only as essential structural components but also as initiators of the granuloma formation process. In addition, βc cytokines are recognized as vital growth factors that regulate macrophage differentiation and function. Our in-depth analysis of human transcriptomic datasets from sarcoidosis patients has highlighted a significant role for βc cytokine signaling across multiple datasets. The results from our comprehensive prophylactic and therapeutic treatment regimens demonstrated that βc receptor antagonism can effectively disrupt various stages of granuloma initiation and progression *in vitro*. Furthermore, studies utilizing humanized hβcTg mice have provided compelling *in vivo* evidence that anti-βc receptor antibodies significantly reduced granuloma burden in murine models of lung sarcoidosis. Additionally, our thorough transcriptomic analysis of mouse lung tissue suggested that CSL311 has the potential to inhibit cellular metabolism and inflammatory signaling pathways. The current *in vivo* study offers valuable proof of concept, indicating that βc signaling plays a crucial role in the development of vimentin-induced pulmonary granulomas. To strengthen the findings of this research, it would be beneficial to address some limitations. Specifically, the exclusion of Ni-NTA beads-only and matrix-only groups was based on prior validation. Including these groups in future investigations could help eliminate potential bias from bead or matrix effects. In addition, to build on the validated effects of CSL311 observed in this study, further research is needed to elucidate the kinetics of granuloma formation, establishment, and resolution within this model. This understanding will facilitate the rigorous evaluation of CSL311 in a therapeutic context once granulomas have formed. Additionally, it will be essential to further investigate the pathogenesis of sarcoidosis in both male and female mice, ensuring that our findings are comprehensive and applicable to both sexes.

In addition to their roles as growth and activation factors, βc cytokines fulfill important non-growth factor functions that contribute to sarcoidosis pathogenesis. IL-3 and GM-CSF, in particular, are key regulators of lipid metabolism, which could be critical for the function of granuloma macrophages in cases of chronic sarcoidosis (28, 29). Recent investigations have shown that these macrophages exhibit notable abnormalities in lipid metabolism, marked by the accumulation of “foamy” neutral lipids. RNA-seq has shed light on the dysregulation of lipid metabolism in GM-CSF-stimulated monocyte-derived macrophages from chronic sarcoidosis patients. This research highlights the enrichment of pathways and genes associated with lipid metabolism that operate through the mechanistic target of rapamycin (mTOR) signaling pathway (27). The mTOR pathway is crucial for the persistence of granulomas, as it is chronically upregulated in macrophages and plays a significant role in regulating lipid droplet dynamics and cellular responses to changes in lipid availability (30). A recent study found that the mTOR inhibitor sirolimus resulted in clinical improvements, including a decrease in skin granuloma size, with sustained effects observed even after short-term administration (31). Both IL-3 and GM-CSF have been shown to strongly activate the mTOR pathway (32, 33), and our research demonstrates that antagonizing the βc receptor effectively inhibited mTOR pathway activity within granulomas and reduced lipid droplet accumulation in granuloma macrophages. Furthermore, treatment with CSL311 specifically targeted the upregulation of the Lipid and Atherosclerosis pathway, as identified through KEGG-based GSEA, in the lungs of mice treated with vimentin. Genes that directly regulate cholesterol efflux and lipid transport (*Abca1*, *Abcg1*, *Apob* and *Pparg*), genes involved in foam cell formation (*Lbp*, *Ly96*, *Olr1*, *Nox1*, *Cyba*, *Cybb* and *Sod2*) were targeted by βc receptor antagonism (34). These findings elucidated the significant relationship between mTOR activation and lipid droplet accumulation by βc cytokines in sarcoid granulomas. This enhanced understanding not only deepens our knowledge of granuloma formation and persistence but also paves the way for innovative therapeutic strategies through βc antagonism.

Patients with long-standing sarcoidosis face an increased risk of developing pulmonary fibrosis, with reported incidences up to 20% (35). Furthermore, approximately 15% of individuals with pulmonary fibrotic disease may progress to a more severe sarcoidosis phenotype (36). In our current investigation utilizing a vimentin-induced sarcoidosis model, we have successfully identified an enrichment of gene sets associated with lung fibrosis and tissue remodeling in the lung transcriptome following the vimentin challenge. This finding suggests that the vimentin-induced model may closely resemble an advanced stage of sarcoidosis, incorporating the fibrotic characteristics typically observed in progressive disease. Notably, we discovered that βc receptor antagonism significantly reduces the enrichment of these gene signatures. This observation is consistent with our earlier research on asthma and COPD models, where treatment with CSL311 resulted in a marked decrease in lung fibrosis-related gene signatures (15, 17). These results provide valuable insights for further exploration of targeted therapies aimed at improving outcomes for patients with sarcoidosis and related pulmonary fibrosis.

There has been significant interest in targeting GM-CSF signaling in sarcoidosis, given its central role in modulating the function of monocytes/macrophages. This has led to a recent Phase II clinical trial in chronic pulmonary sarcoidosis, RESOLVE-LUNG (NCT05314517), which utilizes Namilumab, an anti-GM-CSF monoclonal antibody (37). However, preliminary data from this recent trial indicated that Namilumab did not achieve its primary endpoints, suggesting that targeting the GM-CSF ligand alone does not provide a treatment benefit for patients with chronic active pulmonary sarcoidosis (38). It is well-documented that the shared βc cytokine family (GM-CSF, IL-3, and IL-5) displays a degree of functional overlap and redundancy in regulating myeloid cell function, including proliferation, survival, phagocytosis, and cytokine production. Our *in silico* analyses also revealed that IL-3 and IL-5 pathways, which signal via the shared βc receptor, are concurrently activated in progressive lung sarcoidosis, with the combined (IL-3, IL-5, and GM-CSF) pathway exhibiting the highest enrichment in both GSEA and GSVA results. This suggests that targeting the shared βc receptor, rather than individual cytokines, may offer broader therapeutic efficacy. Furthermore, GM-CSF levels can fluctuate widely in different tissue niches, and targeting the shared receptor in tissue and on circulating myeloid cells may provide a more effective strategy to reduce signaling through this disease-associated pathway.

Through the integration of data from human transcriptomic data, preclinical models, and therapeutic interventions, our study underscores the pivotal role of βc cytokine signaling in granuloma formation and the progression of sarcoidosis. We demonstrated a promising potential therapeutic strategy aimed at antagonizing the functions of βc cytokines within granulomas, which has the potential to alleviate inflammation. Furthermore, our research provides insights into therapeutic approaches for modulating lipid dynamics in macrophages and preventing fibrotic changes associated with sarcoidosis. This contributes to the advancement of more effective treatment options and therapeutic strategies in this area, indicating a positive direction for future research and clinical applications.

## Data Availability

The datasets presented in this study can be found in online repositories. The names of the repository/repositories and accession number(s) can be found below: https://www.ebi.ac.uk/biostudies/studies?query=S-BSST2277.
