# Supplementary material for: βc receptor antagonism mitigates sarcoidosis granuloma formation by targeting inflammatory signals and aberrant lipid metabolism
Source: Front Immunol. 2025 Dec 16;16:1733060. doi: 10.3389/fimmu.2025.1733060 (PMC12747970; doi:10.3389/fimmu.2025.1733060)
Supplement: Supplementary file 1 [file DataSheet1.pdf]

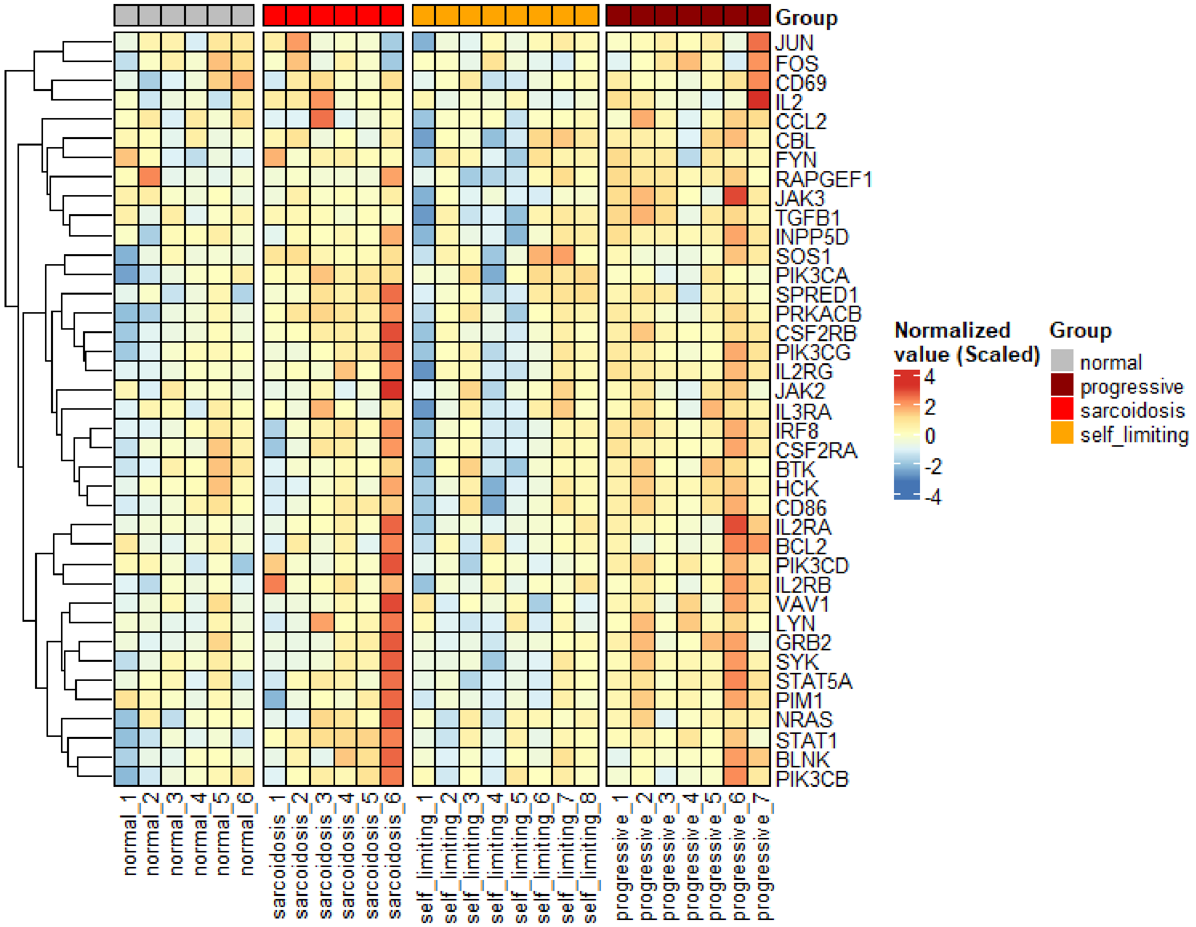

Figure S1: Heatmap visualization of core enrichment genes from pathways relating to βc cytokine signaling across normal lung, sarcoidosis lung, self-limiting sarcoidosis, and progressive sarcoidosis groups. To explore pathway enrichment for βc cytokine signaling, publicly available lung transcriptomic datasets comparing sarcoidosis lung tissues with normal lung tissue (GSE16538, PMID: 20194811) and lung tissues from patients with nodular, self-limiting disease versus those with progressive, fibrotic disease (GSE19976, PMID: 20194811) were explored. The two datasets were combined for co-analysis, and batch effects were removed. GSEA was performed, and the expression level of core enrichment genes for 'Reactome: Interleukin-3, Interleukin-5 and GM-CSF signaling', 'WP: IL3 signaling', 'WP: IL5 signaling', 'PID: GMCSF signaling' are visualised in a heatmap.

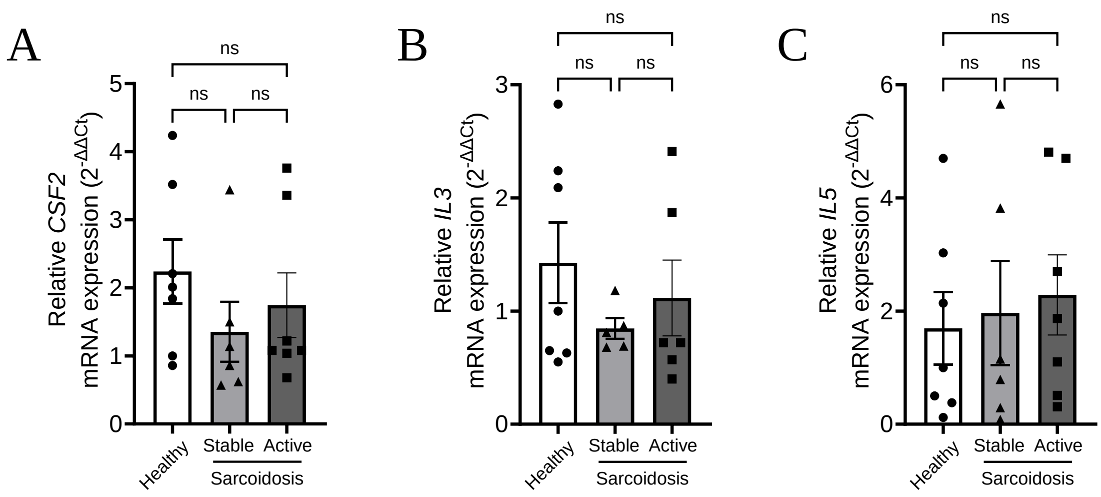

Figure S2: Negligible difference in βc cytokine gene expression in sarcoidosis patient's PBMCs. Transcriptional expression of βc cytokines diseases (A) GM-CSF, (B) IL-3 and (C) IL-5 in PBMCs from healthy donor controls and sarcoidosis patients with stable or active diseases. Data are mean + S.E.M. from 3 -6 donors in each group. One-way ANOVA with Bonferroni post-test.

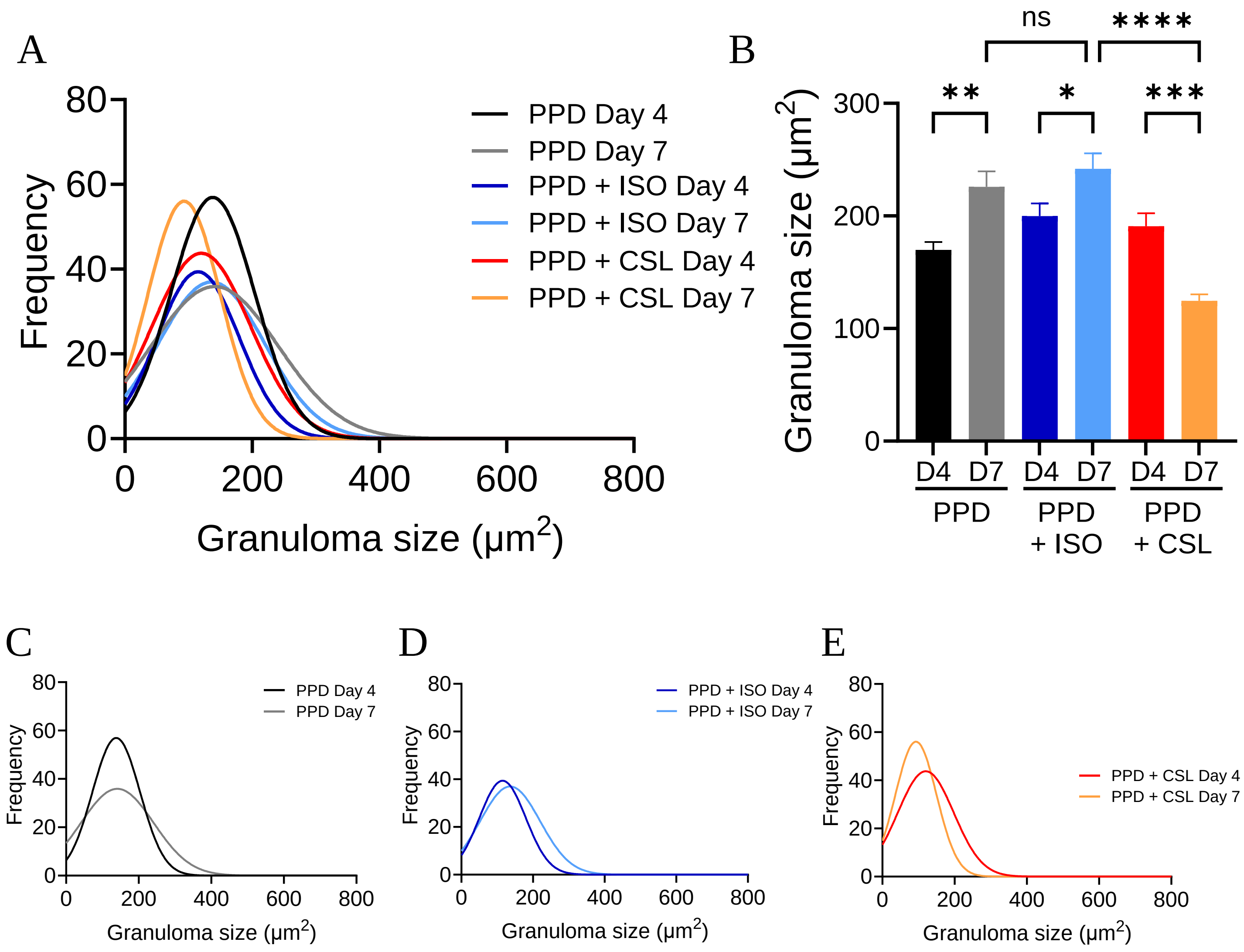

Figure S3: Therapeutic treatment of CSL311 reduced in vitro granuloma size. The size of each individual granuloma was measured and presented in two formats: (A) a frequency distribution graph and (B) a bar graph. (C - E) The size of granulomas for each treatment group at different time points. Data are representative of 7 independent experiments with similar results. ISO – isotype antibody, CSL – CSL311.

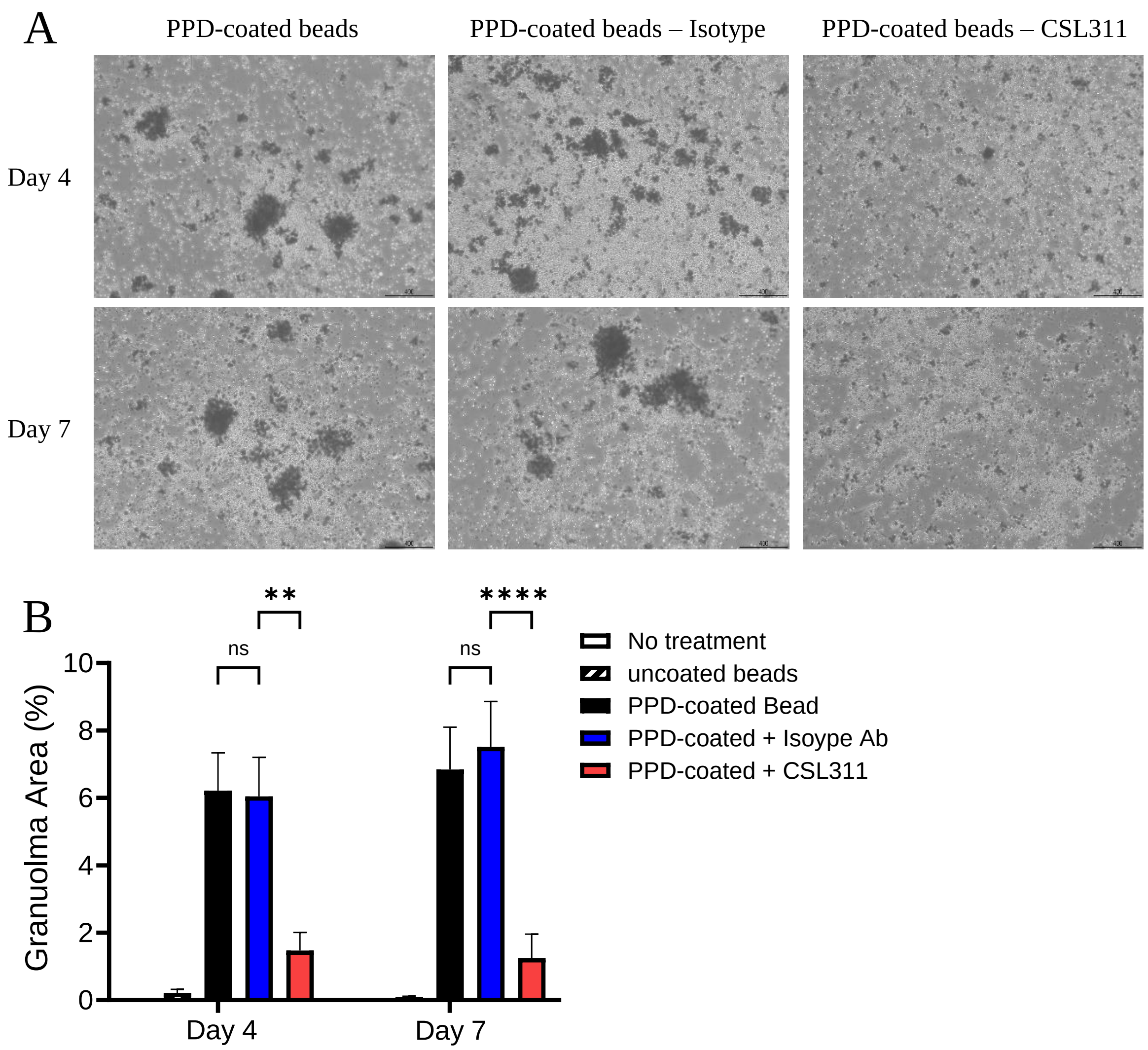

Figure S4: Prophylactic treatment of CSL311 inhibited in vitro granuloma-like aggregate formation. Representative bright field microscopy images (100x magnification) were captured on days 4 and 7 after the incubation of PBMCs with PPD-coated or uncoated beads. The PBMCs were initially incubated with Isotype control mAb or CSL311 (100 µg/ml) for 30 minutes before the addition of PPD-coated beads. The error bar is 200 µm. (B) Quantitative measurement of granuloma area at days 4 and 7 under all treatment conditions. Data are mean + S.E.M of 8 independent experiments. Two-way ANOVA with Bonferroni post-test.

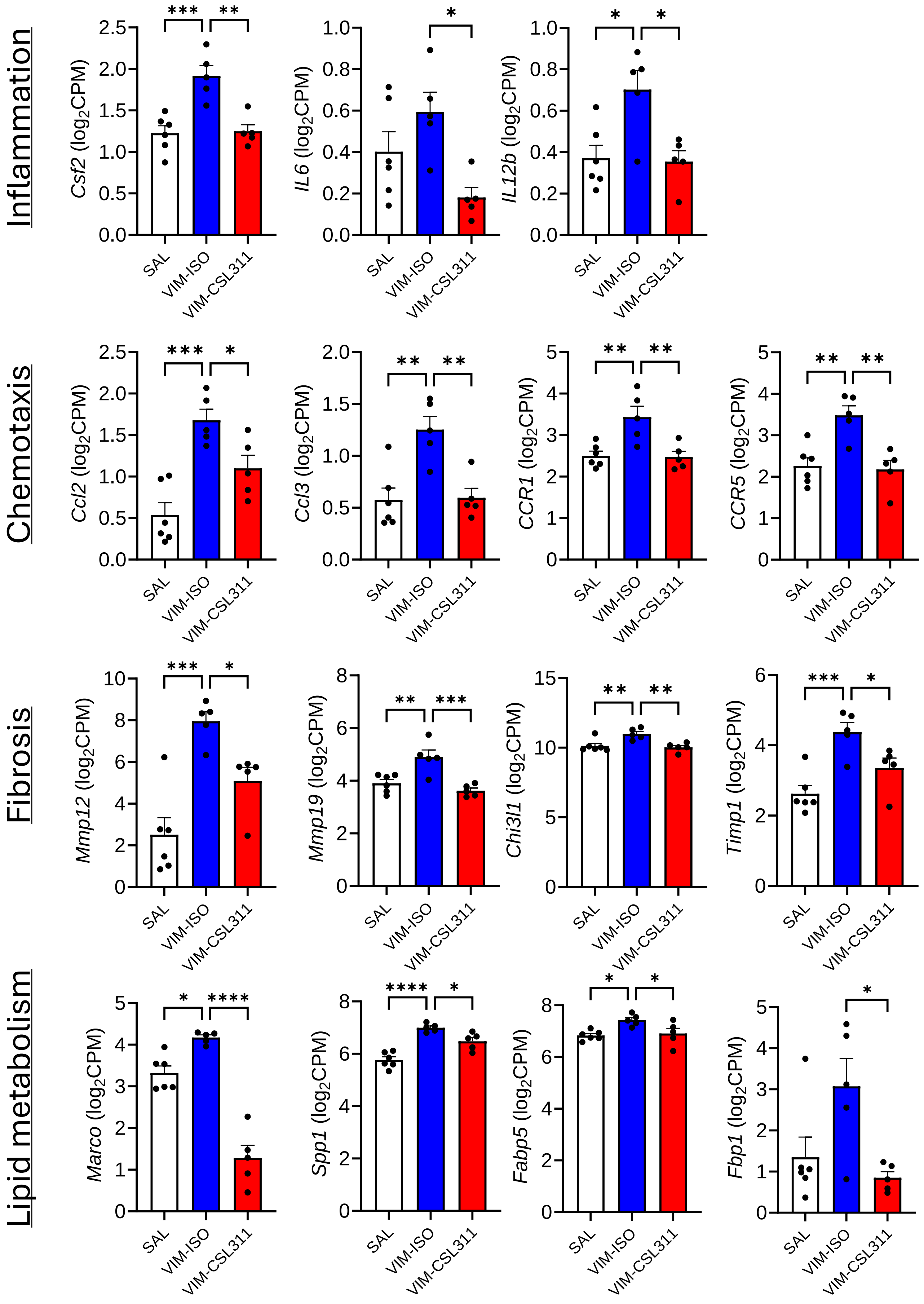

Figure S5. Grouped analysis of key genes involved in vimentin-induced lung sarcoidosis. RNA-seq was performed on lungs from control mice (SAL, n = 6), vimentin-treated mice injected with isotype control (VIM-ISO, n = 5) or CSL311 (VIM-CSL311, n = 5). Key genes involved in granuloma inflammation, immune cell infiltration into granulomas, fibrosis related to sarcoidosis, and lipid metabolism in foamy macrophages were analysed. Data are mean ± S.E.M. One-way ANOVA with Bonferroni post-test.

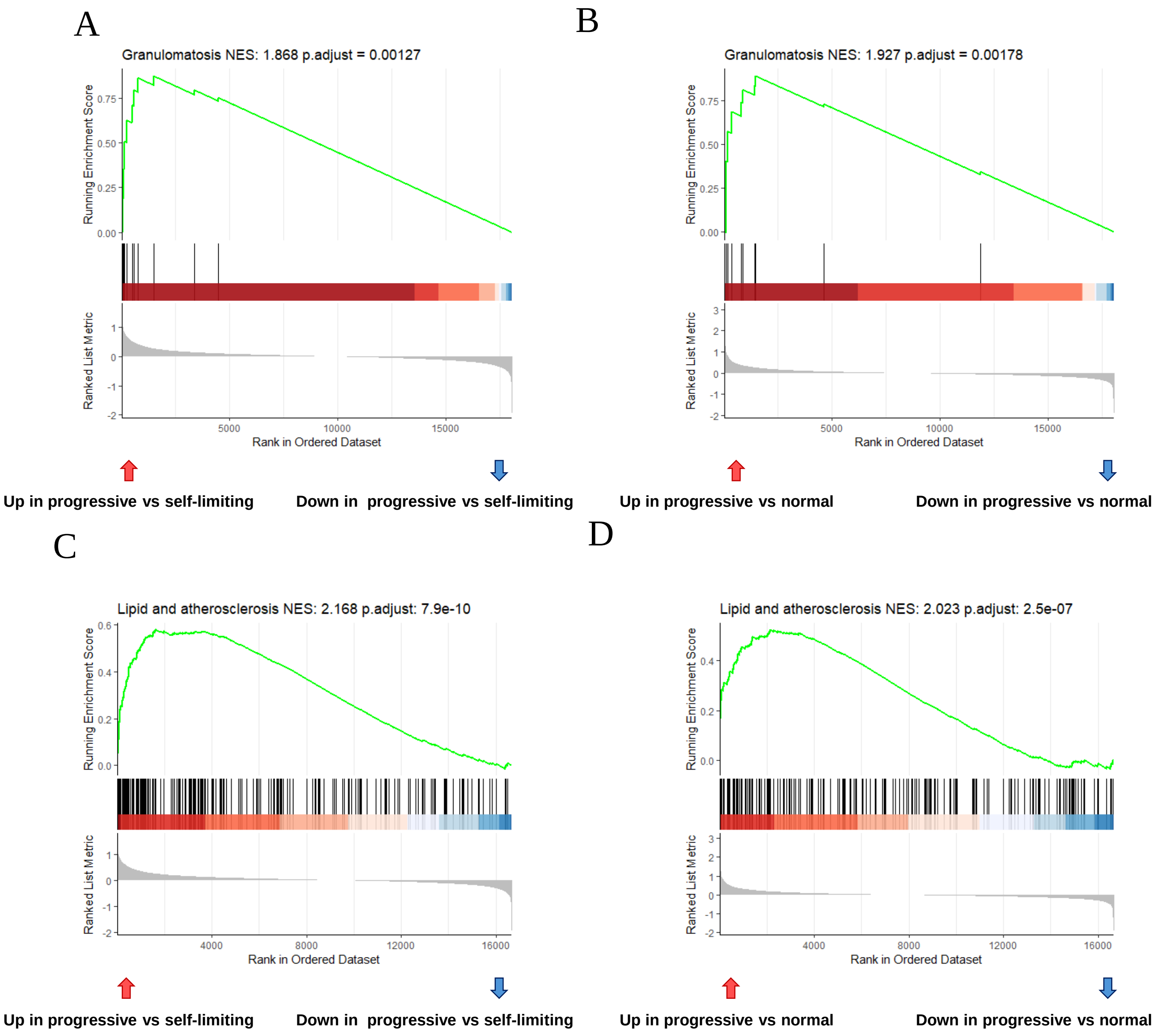

Figure S6: Validation of Granuloma-associated pathways, and the Lipid and Atherosclerosis pathway in human sarcoidosis. To assess the robustness of the Granulomatosis (HP:0002955) from The Human Phenotype Ontology (HPO) database, publicly available lung transcriptomic datasets comparing sarcoidosis lung tissues with normal lung tissue (GSE16538, PMID: 20194811) and lung tissues from patients with nodular, self-limiting disease versus those with progressive, fibrotic disease (GSE19976, PMID: 20194811) were explored. The two datasets were combined for co-analysis, and batch.
