# Supplementary material for: βc receptor antagonism mitigates sarcoidosis granuloma formation by targeting inflammatory signals and aberrant lipid metabolism
Source: Front Immunol. 2025 Dec 16;16:1733060. doi: 10.3389/fimmu.2025.1733060 (PMC12747970; doi:10.3389/fimmu.2025.1733060)
Supplement: Supplementary file 2 [file Table2.docx]

|  | **Tissue Histology** | **BAL cell analysis** | **RNA-sequencing** |
| --- | --- | --- | --- |
| Saline | 12 | 6 | 6 |
| VIM-ISO | 12 | 6 | 5 |
| VIM-CSL | 11 | 6 | 5 |

**Table S2. The allocation of animals for endpoint analysis in the *in vivo* study.** The number reflects the animals utilized for each analysis. It is important to note that one tissue sample from the VIM-ISO group was excluded from RNA sequencing due to a quality control issue.
